# A Monoclonal Antibody-Based Immunochromatographic Test Strip and Its Application in the Rapid Detection of Cucumber Green Mottle Mosaic Virus

**DOI:** 10.3390/bios13020199

**Published:** 2023-01-29

**Authors:** Zichen Zhao, Yanli Tian, Chang Xu, Yuanfei Xing, Lili Yang, Guoliang Qian, Xiude Hua, Weirong Gong, Baishi Hu, Limin Wang

**Affiliations:** 1 Department of Phytopathology, College of Plant Protection, Nanjing Agricultural University, Nanjing 210095, China; 2 Department of Pesticide Science, College of Plant Protection, Nanjing Agricultural University, Nanjing 210095, China; 3 Plant Protection and Quarantine Station of Jiangsu Province, Nanjing 210036, China

**Keywords:** monoclonal antibodies, immunochromatographic strip, cucumber green mottle mosaic virus, sandwich

## Abstract

Two specific monoclonal antibodies (mAbs) were screened, and an immunochromatographic strip (ICS) test for rapid and specific detection of cucumber green mottle mosaic virus (CGMMV) was developed. The coat protein of CGMMV was heterologously expressed as an immunogen, and specific capture mAb 2C9 and the detection mAb 4D4 were screened by an uncompetitive immunoassay. The test and control lines on the nitrocellulose membrane were coated with the purified 2C9 and a goat anti-mouse IgG, respectively, and a nanogold probe combined with 4D4 was applied to the conjugate pad. Using these mAbs, a rapid and sensitive ICS was developed. Within the sandwich mode of 2C9–CGMMV–4D4, the test line showed a corresponding positive relationship with CGMMV in infected samples. The ICS test had a detection limit of 1:5000 (*w*/*v*) for CGMMV in samples and was specific for CGMMV, with no observed cross-reaction with TMV or CMV.

## 1. Introduction

Cucumber green mottle mosaic virus (CGMMV) is a plus-stranded RNA virus and a member of the Tobamovirus genus [1]. CGMMV infects crops, such as cucumber, watermelon and zucchini, causing plant growth retardation, dwarfing, leaf mottling, deformity, fruit yellowing, water damage and other symptoms [2]. The disease caused by CGMMV has become one of the most serious viral diseases in Cucurbitaceae crops because of the rapid spread and difficulty in eradication [3,4]. The Ministry of Agriculture of China added CGMMV to the list of quarantine pests in 2007 because of the potential threat to the production and quality of cucumber crops [5]. In addition, the disease cannot be accurately detected during the early stage of plant growth and is thus easily misdiagnosed, leading to serious outbreaks during the harvest season. Therefore, a fast, accurate and simple detection method for CGMMV is urgently needed.

Currently, many types of assays have been used to detect CGMMV, including biological indexing [6], ELISA [7], one-step reverse transcription loop-mediated isothermal amplification [8], real-time RT-PCR and optical coherence tomography [9]. Although these methods can achieve accurate detection results, they are not suitable for the online screening of samples on a large scale because of complex sample pretreatment requirements, professional operating techniques and lengthy analysis times [10]. Thus, there is a growing requirement for visible, economical and rapid methods that detect CGMMV accurately.

The immunochromatographic assay (ICA) based on the specificity reaction between antigen and antibody was developed for qualitative and quantitative detection and rapid sample screening [11]. Compared with ELISA and other analysis techniques, the ICA method has several advantages, including fast and convenient operation [12], low cost and stable performance [13]. As a result, there is increasingly widespread use of immunochromatographic strips (ICS) for rapid and point-of-care detection of pesticides, heavy metals, toxins, proteins, hormones, pathogens and drugs [14]. For example, an ICA detected tylosin and tilmicosin in milk, where the cut-off value for tylosin was 8 ng/mL [15]. Moreover, an ICS test was used to detect imidacloprid in agricultural and environmental samples with a LOD of 0.45 ng/mL [16]. For in vitro diagnosis, ITS tests can be used to screen for cryptococcal antigens in clinical serum and cerebrospinal fluid specimens [12].

The development and use of immunochromatographic detection methods to detect plant pathogens continue to increase. For example, Bin [17] developed an ICS to detect the citrus yellow vein clearing virus (CYVCV), which was able to detect CYVCV from tissue extracts at a ratio of 1:320 (*w*/*v*). ICS tests have been used to diagnose many plant diseases, including those caused by citrus tristeza virus (CTV) [18], tobacco mosaic virus (TMV) [19] and soybean mosaic virus (SMV) [20]. There are no published reports on colloidal gold ICS for CGMMV detection.

In this study, we used *E. coli* expression of the CGMMV coat protein as an immunogen in mice for the production of monoclonal antibodies (mAbs). Six mAbs were prepared using hybridoma cell lines via cell fusion, and antibodies 4D4 and 2C9 were shown to have the optimum pairing detection effect. The sensitivity of the test strip reached 1:5000 (*w*/*v*), and accurate results were obtained in 10 min. No cross-reaction with other viruses tested was found. This method has good application prospects and is suitable for large-scale sample detection in the field, with low technical requirements for testing personnel and rapid detection. To the best of our knowledge, no ICS for detecting CGMMV has been reported. Based on this ICS, a potential qualitative test method for detecting CGMMV was realized. Establishing an accurate, sensitive and rapid immunoassay system for on-site screening of CGMMV represents a future goal based on the work presented herein.

## 2. Materials and Methods

### 2.1. Reagents

The SP2/0 cell line was stored and cultured by the plant quarantine and bacteriology laboratory of Nanjing Agricultural University. Colloidal gold particles (40 nm in diameter) were prepared using the trisodium citrate reduction method, as described by Contreras-Trigo et al. [21]. Bovine serum albumin (BSA), Tween-20, dimethyl sulfoxide (DMSO), 3,3′,5,5′-tetramethylbenzidine, Freund’s complete/incomplete adjuvants and polyethylene glycol (PEG1500) were purchased from Sigma Chemical Co. (St. Louis, MO, USA). Hypoxanthine aminopterin thymidine (HAT), hypoxanthine thymidine (HT) and Dulbecco’s Modified Eagle Medium (DMEM) were provided by Gibco (Grand Island, NY, USA). Horseradish peroxidase-labeled goat anti-mouse IgG conjugate (HRP-GaMIgG) was acquired from Zhuyan Biological Technology Co., Ltd. (Nanjing, China). Fetal bovine serum (FBS) was provided by Lanzhou Minhai Biological Engineering Co., Ltd. (Lanzhou, China).

### 2.2. Equipment

Nitrocellulose membranes, absorbent pads, glass fibers and PVC plates were purchased from Millipore Corp (Billerica, MA, USA). An XYZ-3060 dispensing platform and CM4000 Guillotine Cutter (BioDot, Irvine, CA, USA) were used to prepare test strips. A membrane strip reading instrument (TSR5000) was purchased from Jiening Biotech Co., Ltd. (Shanghai, China). A Forma Series II CO_2_ incubator was supplied by Thermo Electron (Waltham, MA, USA). A vacuum drying oven was purchased from Shanghai Senxin Experimental Instrument Co., Ltd. (Shanghai, China). A dehumidifier was purchased from Zhuyan Biological Co., Ltd. (Nanjing, China).

BALB/c female mice were purchased from the Center of Comparative Medicine of Yangzhou University (Yangzhou, China).

### 2.3. Preparation of the Immunogen

#### 2.3.1. Source of Virus

Tobacco mosaic virus (TMV), cucumber mosaic virus (CMV) and cucumber green mottle mosaic virus (CGMMV) on different hosts used in the experiments were preserved by the plant quarantine and bacteriology laboratory of Nanjing Agricultural University.

#### 2.3.2. Virus Inoculation

Virus inoculation was processed according to Murphy [22] and Stefanov et al. [23], with minor modifications. Tobacco leaves (1 g) infected with CGMMV were ground thoroughly in a sterile mortar with an appropriate amount of inoculation buffer (PBS, 0.01 M, pH 7.0). Tobacco plants growing at 3rd or 4th leaves were mechanically inoculated on carborundum-dusted 1st and 2nd true leaves with abrasive fluid. The mock-inoculated plants were rubbed in the same manner but with buffer only. Healthy leaves with similar growth status were inoculated with a vaccination buffer as a negative control at the same time. Inoculated plants were placed in a greenhouse and closely observed for growth and morbidity. Plants began to show weak symptoms 7–10 days after inoculation, and the leaves showed slight shrinkage, with clear symptoms of virus infection appearing after approximately 14 days. At this time, the infected and healthy leaves were harvested and stored at −80 °C for later use.

#### 2.3.3. Immunogen Preparation

The immunogen was prepared by the plant virology laboratory of Nanjing Agricultural University. Preparation was carried out according to Li [8] and Su [24], with minor modifications. Total RNA was extracted from CGMMV-infected leaves using Trizol. Genomic DNA was removed, and RNA was reverse transcribed into cDNA using 5× Hiscript qRT Supermix. A pair of specific primers were designed based on the reported CGMMV coat protein gene sequence (Accession number: EU366912.1): upstream primer (ZC1F), CGCGGATCCATGGcttacaatccgatcacaccta; downstream primer (ZC2R), CCGCTCGAGCTAagctttcgaggtggtag. The upstream and downstream primers included BamHI and XhoI digestion sites, respectively. The digested fragment was ligated into the pET28a vector using the BamHI and XhoI sites to form a recombinant vector. The recombinant vector was purified and transformed into *E. coli* (Rosta) for protein overexpression. The purified protein was used as an immunogen of CGMMV [25,26].

### 2.4. Preparation of the Monoclonal Antibody against CGMMV

The purified immunogen was diluted with PBS (0.15 M, pH 7.0) to a concentration of 2 mg/mL. The first immunization mix combined the immunogen with an equal amount of Freund’s complete adjuvant. After fully emulsifying the mixture, five BALB/C female mice, aged 6–8 weeks old, were immunized, and each mouse was injected with 200 µL. Each subsequent immunization used a combination of immunogen with Freund’s incomplete adjuvant. The second immunization took place three weeks after the first immunization, and thereafter each immunization interval was two weeks. A total of five immunizations were performed. Starting from the third immunization, 7–10 days after each immunization, antiserum was obtained by taking blood from the tail vein of the mouse and the antibody titer in the serum tested by ELISA. The mouse with the highest serum immune response was selected as a spleen donor for subsequent experiments.

One week before the cell fusion experiment, SP2/0 cells were resuscitated and cultured in DMEM medium supplemented with 20% FBS. Three days before the cell fusion experiment, the mouse, selected as the spleen donor, was intraperitoneally injected with 200 µL of immunogens without the immunization adjuvant for booster immunization. At the same time, the mouse spleen was removed, and spleen cells were obtained after grinding and centrifugation. The spleen cells and SP2/0 myeloma cells were mixed evenly. PEG1500 was added at a uniform rate within one minute for the cell fusion test, and DMEM was used to terminate the reaction. Fused cells with complete culture medium were suspended and evenly spread into 96-well cell plates. Fused cells were cultured in a 5% CO_2_ incubator at 37 °C, and after seven days, with 3,3′,5,5′-tetramethylbenzidine as the main component of the indirect ELISA, positive pores were screened. The cells were screened by HAT and HT screening, subcloned by multiple limited dilution methods, and hybridoma cells with high titer were obtained. After three subcloning rounds, monoclonal cell lines capable of stably producing an antibody were obtained. The complete culture solution, bovine serum and DMSO were used to prepare the cryopreservation solution according to a certain proportion, and the selected monoclonal cells were frozen in a liquid nitrogen tank. Mouse ascites collected were purified using an antibody affinity chromatography column to obtain a higher purity mAb, which was stored at −80 °C. Unpurified ascites mixed with 50% glycerol were frozen at −20 °C.

### 2.5. Preparation of the Nanogold–mAb Probe

The preparation of the nanogold probe combined with the mAb is based on the process by Byzova et al. [27], with minor modifications. Initially, the pH of the colloidal gold solution was adjusted to 8.2 with 0.1 M K_2_CO_3_, and 3.2 mg/mL of the solution was added to 10 mL of the pH-adjusted colloidal gold solution. The solution was then mixed vigorously for 5 min and left standing at room temperature for 2 h. The addition of the mAb should be 10% more than the optimal reference amount. Filter-sterilized 10% BSA was then added to a final concentration of 1% and rapidly mixed for 5 min. After standing at room temperature for 1.5 h, the mixture was centrifuged (10,000 rpm) at 4 °C for 30 min, and the supernatant was removed carefully. Finally, the obtained sediment was suspended with 0.02 M Tris-HCl buffer containing BSA (1.0%), sucrose (1.0%) and Tween-20 (0.25%) and stored at 4 °C for future study in 20 days.

### 2.6. Assembly of the ICS Test

**Figure 1:**
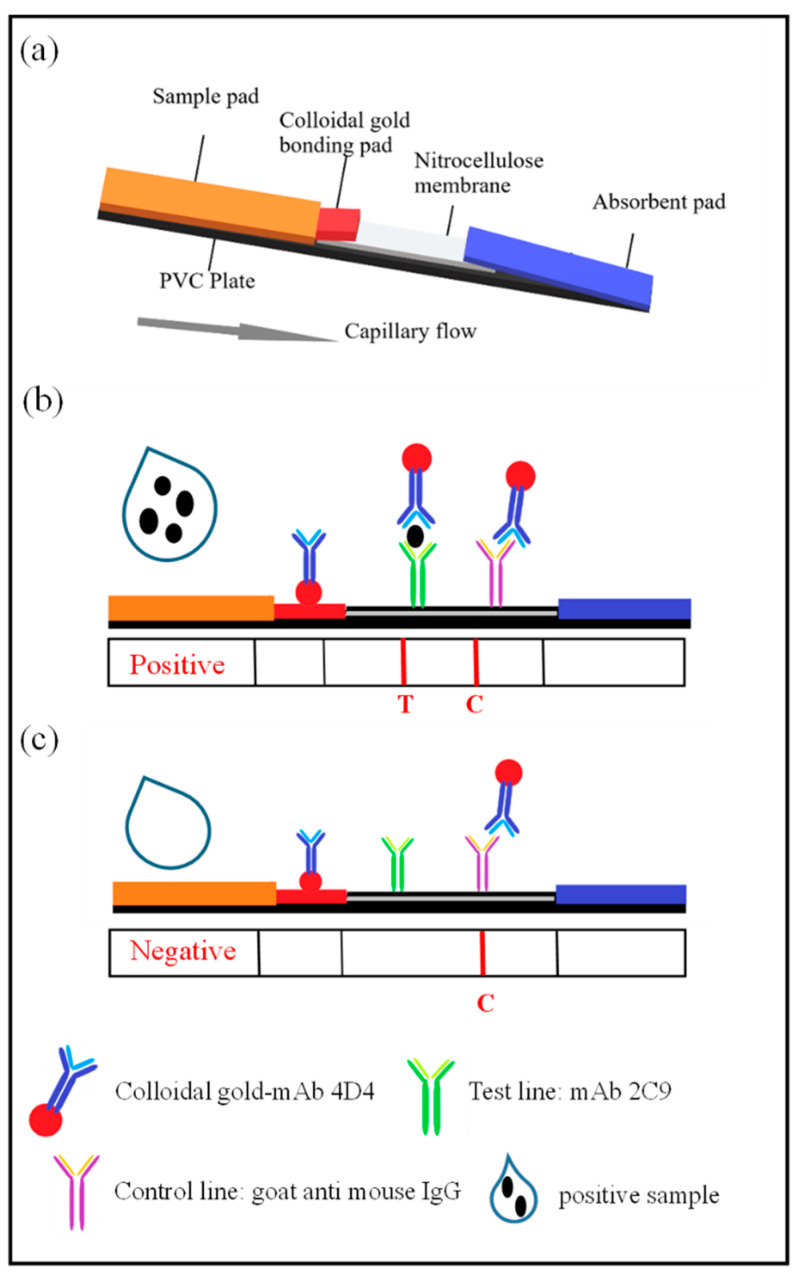
Characterization of the test results. (**a**) Schematic diagram of the test strip structure. (**b**) The reaction that occurs with the mAbs 4D4–2C9 in the presence of the CGMMV antigen. (**c**) In the absence of the CGMMV immunogen in the sample, the reaction only occurs on the control (C) line.

The ICS test consists of five parts: a sample pad, a bonding pad, a nitrocellulose membrane, an absorbent pad and a PVC plate (Figure 1a). The nanogold–mAb probe was evenly distributed onto the bonding pad (containing 7.5% EDTA) using XYZ-3060 and dried. The sample pad was treated with 0.01 M PBS containing 0.2% Tween-20. The capture antibody (containing 3% sucrose) was diluted with 0.01 M PBS and evenly sprayed onto the T line, while 0.5 mg/mL of the goat anti-mouse IgG dilution was evenly dispensed onto the C line. The nitrocellulose membrane was placed in a vacuum oven at 37 °C and vacuum dried for 50 min. The distance between the T and the C lines was approximately 5 mm. The sample, bonding and absorbent pads and nitrocellulose membrane were assembled, attached to the PVC plate in the correct order and cut into 3-mm wide test strips using a CM4000 slitter. Prepared test strips were sealed in a plastic bag with a desiccant, vacuumed dried and stored at room temperature.

### 2.7. The Immunochromatographic Assay

In the ICS assay, a 100 µL liquid sample was placed onto the sample pad. The sample then migrates by capillary action through the test strip. Samples containing CGMMV particles react with nanogold-combined 4D4 in the colloidal gold bonding pad. The nanogold–4D4–CGMMV complex then migrates into the nitrocellulose membrane and reacts with mAb 2C9 on the T line to form the nanogold-combined 4D4–CGMMV–2C9 sandwich complex, which is visibly observed as a red band. The nanogold–4D4 combination without CGMMV particles migrates past the T line and reacts with goat anti-mouse IgG on the C line to form the nanogold–4D4–IgG complex, producing the second visible red band (Figure 1b,c). The remaining part of the solution moves along the strip and finally accumulates in the absorption pad. In tested samples that do not contain CGMMV, the T line does not develop a color, but the C line typically develops a color.

### 2.8. Optimization of the ICS

#### 2.8.1. Optimization of the Nitrocellulose Membrane

In the process of test strip assembly and testing, we found that the type of nitrocellulose membrane used affected the color development of the test strip significantly. Therefore, five different nitrocellulose membranes were tested while keeping other test parameters constant (Table S1). The most suitable material was selected as the final condition of the test strip.

#### 2.8.2. Optimization of the Buffer System Parameters for the ICS

The chromatographic buffer of the test strip is very important. An appropriate buffer solution system ensures that the test strip chromatography process proceeds rapidly without a strong background and clearly visible detection of the line(s). In this study, the buffer system was optimized according to the ionic strength, buffer pH and the amount of surfactant. The optimal buffer was selected according to the chromatographic effect and test accuracy of the test strip under different conditions.

## 3. Results

### 3.1. Preparation of the Monoclonal Antibodies

After cell fusion and subcloning, six hybridoma cell lines that stably secrete the CGMMV monoclonal antibody were screened. After antibody subtype identification, mAbs 1B10, 4B2, 4D4 and 6D5 were IgG1, and mAbs 2C9 and 6F1 were IgG2a. All light chains were kappa chains. The mAbs titer was tested via an indirect ELISA (Figure S1).

### 3.2. Pairing of the Monoclonal Antibodies

**Figure 2:**
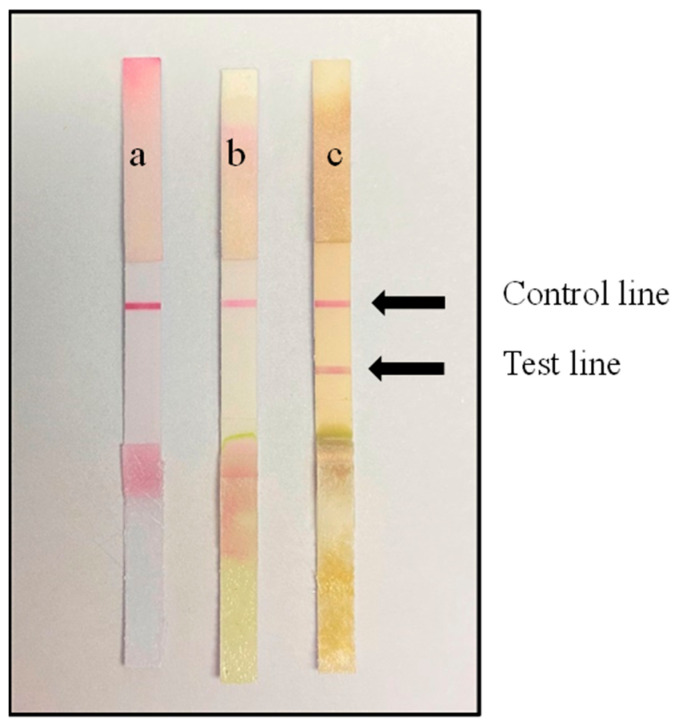
The successful detection of CGMMV using the developed test strip. (**a**) The buffer (0.05 M PBS) chromatography result is negative. (**b**) The test result of healthy cucumber leaves is negative. (**c**) The test result of CGMMV-inoculated cucumber leaves is positive.

After antibody pairing experiments, mAbs 4D4 and 2C9 were paired successfully for the subsequent development of the monoclonal ICS test (Figure 2). Paired antibody detection showed that the T line was present in diseased samples but absent in healthy leaves or buffer solution samples. Therefore, mAbs 4D4 and 2C9 can be used in immunoassay test strips for subsequent testing and optimization.

### 3.3. Optimization of the ICS

#### 3.3.1. Optimization of the Nitrocellulose Membrane

Color development of test strips using five different nitrocellulose membranes (CN95, FF120, Paul 120, CN140, Paul 170) was tested (Table S1). The results showed that no T line was observed for the buffer solution and healthy leaves, whereas a clear T line was observed for samples of diseased leaves. The background signal of the test strip was weak after chromatography, facilitating straightforward observation of the test line. Therefore, we selected Paul 120 as the nitrocellulose membrane for this test strip.

#### 3.3.2. Optimization of Buffer System Parameters for the ICS

##### Influence of Ionic Strength

PBS buffer over a range of different concentrations (0.01–0.15 M) was tested for its effect on the assay when preparing samples. PBS at a concentration of 0.03 M in the test samples yielded the best result, with no false positives observed (Table 1). Therefore, 0.03 M PBS was selected as the working buffer.

##### Influence of pH

PBS buffer over a range of pH values (3.0–9.0) was used to prepare samples, which were then tested with the ICS. False-positive results were not observed on the strip when the buffer pH was ≥6.0. Moreover, the line color intensity was strongest when the pH of the inoculated sample was 6.0 (Table 1). Thus, pH 6.0 was considered the most appropriate buffer pH for the assay.

##### Influence of Tween-20

As a non-ionic surfactant, Tween-20 can reduce the non-specific binding of the antibody without denaturing the protein. In addition, an appropriate amount of Tween-20 can enhance the capture of gold-labeled antibodies on the nitrocellulose membrane. The mAbs were sensitive to Tween-20 because increasing the content of Tween-20 deepened the color of the positive line, enabling the identification of false-positive results. No false-positive effect was observed when the content of Tween was 0.1%, and the color of the line remained sufficiently intense for detecting positive samples (Table 1). Therefore, 0.1% Tween-20 was added to the working buffer.

In summary, PBS with an ionic strength of 0.03 M, 0.1% Tween-20 and pH 6.0 (30 µL of 0.1 M HCl per 1 mL of PBS) was optimal for ensuring the best sensitivity and was selected as the working buffer.

### 3.4. Specificity Assessment of the ICS Test

**Figure 3:**
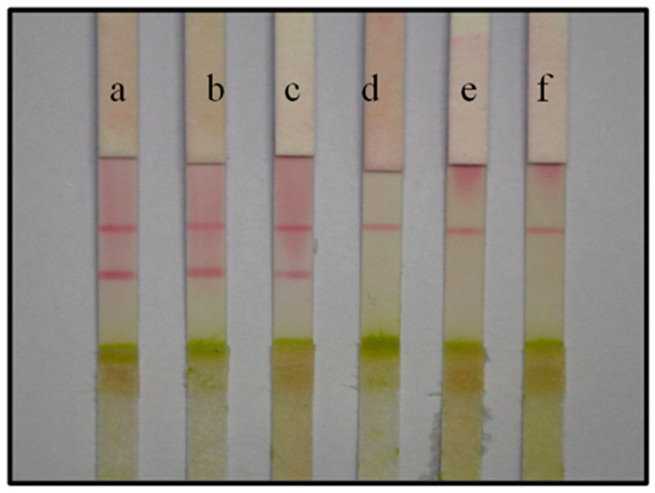
Specificity of the ICS test for detecting CGMMV. (**a**) CGMMV infection of watermelon tested positive. (**b**) CGMMV infection of zucchini tested positive. (**c**) CGMMV infection of tobacco tested positive. (**d**) TMV infection of tobacco tested negative. (**e**) CMV infection of tobacco tested negative. (**f**) A healthy leaf (control) gave a negative result.

Specificity is a critical aspect when detecting CGMMV. Here, we tested CGMMV on Shanxi watermelon and zucchini alongside TMV and CMV. The results showed that the C line and T line of the tested samples from the different host plants infected by CGMMV developed a color response, whereas samples from leaves infected with TMV and CMV showed no color at the T line. As expected, the C line developed for all three virus samples (Figure 3). Therefore, no cross-reaction with TMV and CMV was observed, and the prepared immunochromatographic test strip for detecting CGMMV has acceptable specificity.

### 3.5. Stability Assessment of the ICS Test

**Figure 4:**
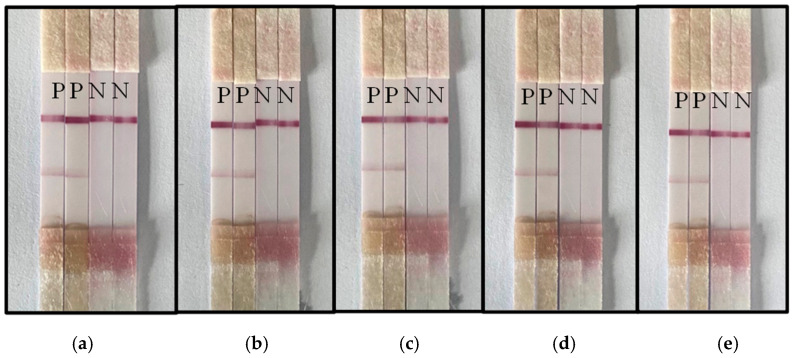
The stability of the test strip for detecting CGMMV. Vacuum storage for (**a**) 14 days, (**b**) 28 days, (**c**) 42 days, (**d**) 56 days and (**e**) 152 days. (P represents CGMMV infected leaves, N represents healthy leaves).

ICS tests prepared in the same batch were placed under a vacuum to dry at room temperature, and the stability of the strips was tested at intervals of 14, 28, 42, 56 and 152 days. Four test strips were randomly selected from the test strips stored for different periods. Two strips were used to test healthy leaves, and the other two were used to test diseased leaves. The results showed that the test strip maintained accurate detection of healthy and diseased leaves after storage under vacuum for an extended period. Therefore, the test strip has acceptable stability (Figure 4).

### 3.6. Sensitivity Assessment of the ICS Test

**Figure 5:**
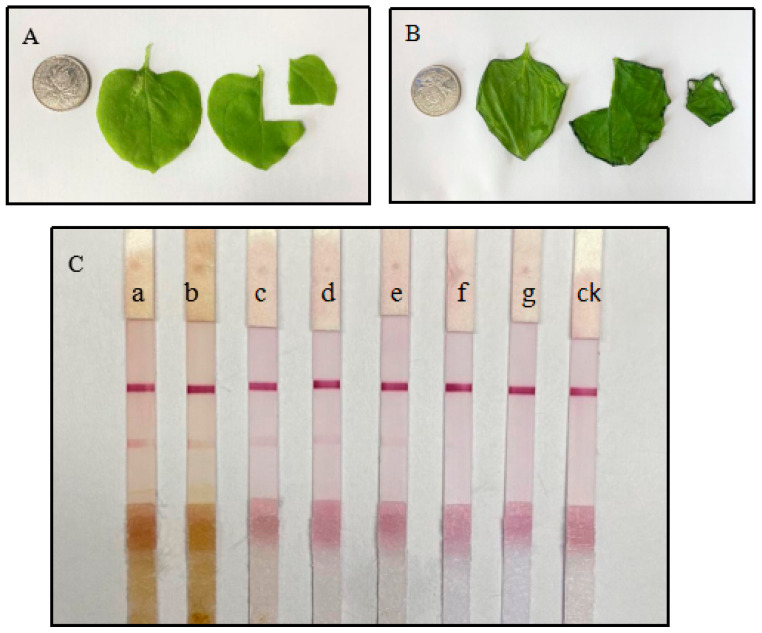
(**A**) Healthy tobacco leaves. (**B**) Inoculated CGMMV tobacco leaves. (**C**) The sensitivity of the test strip to detect CGMMV (disease sample quality, w, and buffer dilution ratio, v). (a) 1:1, (b) 1:10, (c) 1:100, (d) 1:500, (e) 1:1000, (f) 1:5000, (g) 1:10,000. ck: the healthy leaf was negative. Coin-sized samples were ground with 1 mL working buffer for ICS testing.

A series of tobacco leaf slurry concentrations (*w*/*v* ratios ranging from 1:1 to 1:10,000) infected with CGMMV were mixed with the optimized working buffer, and CGMMV detection was tested using the ICS assay. The intensity of the T line decreased as the dilution ratio of the infected leaf slurry increased and was not observed at the highest dilution (Figure 5).

### 3.7. Accuracy of the Test Strip (Field Sample Testing)

**Figure 6:**
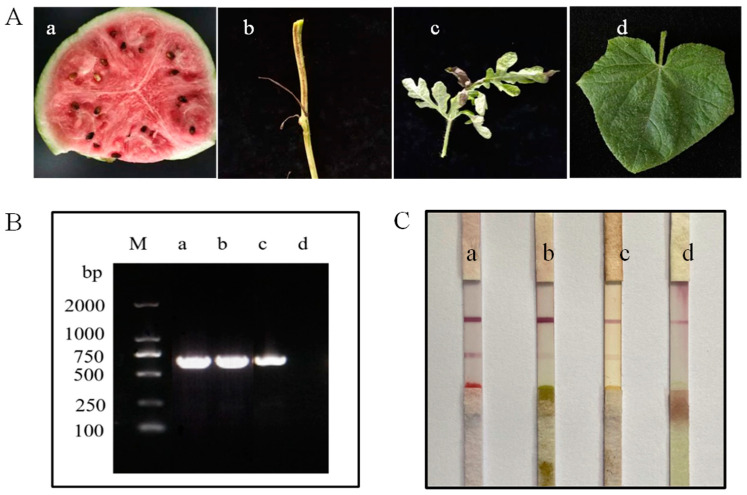
(**A**) Field sample. a: Field-diseased watermelon; b: Field-diseased stem; c: Field-diseased leaf; d: Field-healthy leaves. (**B**) PCR detection of field samples. (**C**) Detection using the ICS test.

The accuracy of the ICS test was evaluated by taking watermelon field samples from Funing County, Yancheng City, Jiangsu Province. Watermelon rattan and leaves were collected. Plant samples of about 1 × 1 cm were placed into a grinding bag, 1 mL working buffer was added, and the sample was gently ground until the grinding solution changed color. Three to four drops of the ground solution were applied to the test strip, and a change in color was monitored. Samples were also taken for PCR analysis. The detection results using the two methods were consistent for diseased and healthy samples. Both assays detected the diseased sample but did not detect the healthy sample (Figure 6).

## 4. Conclusions

A qualitative ICS test for detecting CGMMV was developed, and the detection limit of the test strip was 1:5000 (*w*/*v*). The developed detection assay has excellent specificity and stability. The ICS test based on the sandwich of two monoclonal antibodies displayed high detection accuracy when used to evaluate field samples, which have a more complex biological environment, such as the presence of saprophytic bacteria and pesticide residues. The colloidal gold ICS test provides test results within 10 min, which is much faster than traditional PCR (1–2 h), ELISA (1–2 h) or other detection methods. Thus, the CGMMV ICS test has considerable potential in practical applications. This method is not only suitable for the detection of a single sample but also suitable for the rapid detection of large quantities of samples. Therefore, it is expected to have good application prospects in CGMMV detection and prevention.

## Figures and Tables

**Table 1:** Effect of different buffer parameters on the test strip results.

Optimization Factor		Buffer Solution	Healthy Leaves	Diseased Leaves
PBS	0.01 M	-	-	++
	0.03 M	-	-	+++
	0.05 M	-	-	++
	0.10 M	-	+	+++
	0.15 M	-	+	+++
pH	3.0	+	+	+++
	4.0	+	+	+++
	5.0	+	-	+++
	6.0	-	-	+++
	7.0	-	-	++
	8.0	-	-	++
	9.0	-	-	++
Tween-20 (%)	0.03	-	-	-
	0.05	-	-	+++
	0.10	-	-	++++
	0.15	+	+	++++

“+” represents the color depth of the test strip detection line; ++++ indicates the darkest color of the detected line; +++ is darker than ++; ++ is darker than +; - represents no color.

## Data Availability

The data presented in this study are available on request from the corresponding author. The data are not publicly available due to ethical constraints.

## Supplementary Materials

The following supporting information can be downloaded at https://www.mdpi.com/article/10.3390/bios13020199/s1. Table S1: Effect of different types of nitrocellulose membranes on test strips. Figure S1: Determination of titer of mAbs by indirect ELISA. (negative is incubated with the same amount of healthy leaf grinding solution, and the results are determined by ELISA).
