# Supplementary material for: A Monoclonal Antibody-Based Immunochromatographic Test Strip and Its Application in the Rapid Detection of Cucumber Green Mottle Mosaic Virus
Source: Biosensors (Basel). 2023 Jan 29;13(2):199. doi: 10.3390/bios13020199 (PMC9953337; doi:10.3390/bios13020199)
Supplement: Supplementary file 1 [file biosensors-13-00199-s001.zip › biosensors-2098629-supplementary.pdf]

biosensors MDPI

Supplementary Material

A Monoclonal Antibody-Based Immunochromatographic Test Strip and Its Application in the Rapid Detection of Cucumber Green Mottle Mosaic Virus

Zichen Zhao [1], Yanli Tian [1], Chang Xu [1], Yuanfei Xing [1], Lili Yang [1], Guoliang Qian [1], Xiude Hua [2], Weirong Gong [3], Baishi Hu [1] and Limin Wang [1,*]

*Corresponding author: E-mail address: wlm@njau.edu.cn

Table S1. Effect of different types of nitrocellulose membranes on test strips

Material	Type name	Buffer solution	Healthy leaves	Diseased leaves
nitrocellulose membrane	CN-95	-	-	++
	FF-120	+	+	++++
	Paul-120	-	-	+++
	CN-140	-	-	++
	Paul-170	-	-	+

"+"represents the color depth of the test strip detection line; ++++ indicates the darkest color of the detection line; +++is darker than++; ++is darker than+; -represents that the detection line has no color.

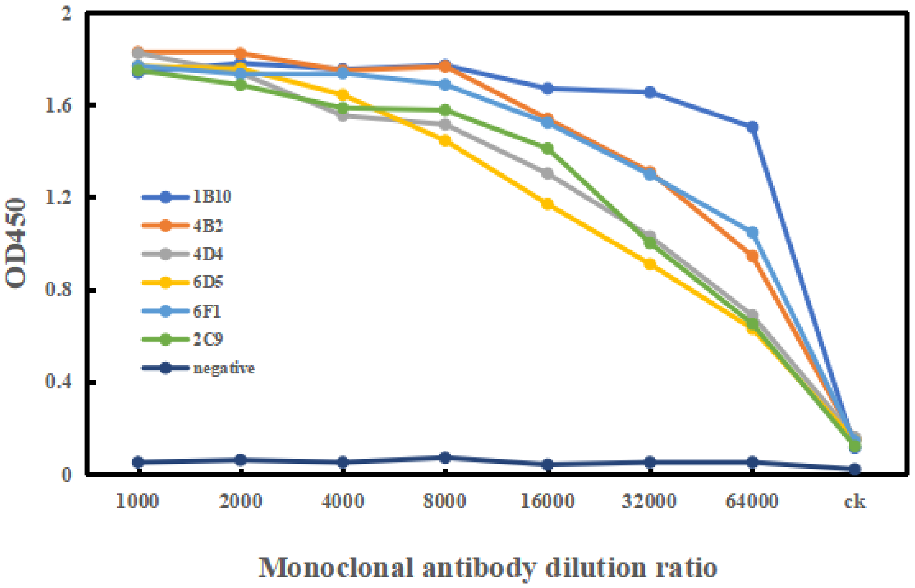

Figure S1. Determination of titer of mAbs by indirect ELISA. (negative is incubated with the same amount of healthy leaf grinding solution, and the results are determined by ELISA).
